# Anaesthetic injection versus ischemic compression for the pain relief of abdominal wall trigger points in women with chronic pelvic pain

**DOI:** 10.1186/s12871-015-0155-0

**Published:** 2015-12-01

**Authors:** Mary L. L. S. Montenegro, Carolina A. Braz, Julio C. Rosa-e-Silva, Francisco J. Candido-dos-Reis, Antonio A. Nogueira, Omero B. Poli-Neto

**Affiliations:** 1 Department of Gynecology and Obstetrics, Ribeirão Preto Medical School of University of Sao Paulo, Bandeirantes Avenue, 3900, Campus Universitário s/n. Monte Alegre, Ribeirão Preto, SP CEP 14048-900 Brazil; 2 Department of Cardiology, Federal University of São Paulo, São Paulo, Brazil

**Keywords:** Chronic pelvic pain, Ischemic compression, Local anaesthetic injection, Trigger point, Abdominal wall, Myofascial syndrome

## Abstract

**Background:**

Chronic pelvic pain is a common condition among women, and 10 to 30 % of causes originate from the abdominal wall, and are associated with trigger points. Although little is known about their pathophysiology, variable methods have been practiced clinically. The purpose of this study was to evaluate the efficacy of local anaesthetic injections versus ischemic compression via physical therapy for pain relief of abdominal wall trigger points in women with chronic pelvic pain.

**Methods:**

We conducted a parallel group randomized trial including 30 women with chronic pelvic pain with abdominal wall trigger points. Subjects were randomly assigned to one of two intervention groups. One group received an injection of 2 mL 0.5 % lidocaine without a vasoconstrictor into a trigger point. In the other group, ischemic compression via physical therapy was administered at the trigger points three times, with each session lasting for 60 s, and a rest period of 30 s between applications. Both treatments were administered during one weekly session for four weeks. Our primary outcomes were satisfactory clinical response rates and percentages of pain relief. Our secondary outcomes are pain threshold and tolerance at the trigger points. All subjects were evaluated at baseline and 1, 4, and 12 weeks after the interventions. The study was conducted at a tertiary hospital that was associated with a university providing assistance predominantly to working class women who were treated by the public health system.

**Results:**

Clinical response rates and pain relief were significantly better at 1, 4, and 12 weeks for those receiving local anaesthetic injections than ischemic compression via physical therapy. The pain relief of women treated with local anaesthetic injections progressively improved at 1, 4, and 12 weeks after intervention. In contrast, women treated with ischemic compression did not show considerable changes in pain relief after intervention. In the local anaesthetic injection group, pain threshold and tolerance improved with time in the absence of significant differences between groups.

**Conclusion:**

Lidocaine injection seems to be better for reducing the severity of chronic pelvic pain secondary to abdominal wall trigger points compared to ischemic compression via physical therapy.

**Trial registration:**

ClinicalTrials.gov NCT00628355. Date of registration: February 25, 2008.

**Electronic supplementary material:**

The online version of this article (doi:10.1186/s12871-015-0155-0) contains supplementary material, which is available to authorized users.

## Background

Chronic pelvic pain (CPP) is a common clinical condition among women of reproductive age [1], with a negative impact on the quality of life [2] and significant socioeconomic repercussions [3]. There are great difficulties in establishing the primary cause of CPP and in proposing adequate treatment. The conditions that are most commonly diagnosed in women with CPP are constipation, irritable bowel syndrome, interstitial cystitis/painful bladder syndrome, endometriosis, and adhesions [4]. In contrast, 10 to 30 % of cases may be originating from the trigger points in the abdominal wall muscle [5]. This condition is usually associated with trigger points, which are usually defined as hyper-irritable sites located within a taut band of skeletal muscle or fascia. When compressed, these trigger points cause referred pain, local tenderness, and autonomic changes [6]. Despite the variability of the criteria used to diagnose them, they can be located easily by trained observers [7]. Some authors have associated them with the abdominal myofascial pain syndrome [8]. However, it may be almost impossible to distinguish between a true nerve entrapment and a myofascial trigger point in the rectus abdominis muscle [7]. Furthermore, they may also be associated with a visceral pain origin [9] such as endometriosis, especially when associated with allodynia [8]. Although little is known about the pathophysiology of the condition, the use of a local anaesthetic has been recommended as an effective technique for the treatment of symptomatic active trigger points [10], including abdominal ones [11]. However, few studies have prospectively evaluated the effects of anaesthetic injections into abdominal wall trigger points [12] and the clear advantages of this procedure has not been established [13]. Among others, ischemic compression, has been identified as a useful non-invasive method for the treatment of trigger points [14]. However, it remains unknown if ischemia actually occurs with this intervention. In our country, non-pharmacological modalities have also been widely used. These are usually preceded by transcutaneous electric nervous stimulation (TENS), only for initial analgesia, as reported in the literature [15]. To our knowledge, there is no currently available data that shows the superiority of any one method. Thus, the objective of the present study was to evaluate the efficacy of local anaesthetic injections versus ischemic compression for pain relief of abdominal trigger points in women with CPP.

## Methods

### Design

This study was a parallel group randomized controlled trial, which utilized blinded outcome assessors, and an intention-to-treat analysis. After meeting the eligibility criteria for the study, participants were randomly allocated by the primary researcher to two experimental groups according to a computer-generated block randomization.

### Registration

The study followed the Declaration of Helsinki and was approved by our Research Ethics Committee (process number:10272/2007, University Hospital, Ribeirão Preto Medical School). All subjects signed the informed consent prior to participation. This study was registered on ClinicalTrials.gov- NCT00628355 on date February 25, 2008.

### Setting

Center of Chronic Pelvic Pain and Gynecologic Endoscopy of the Universitary Hospital, Ribeirão Preto Medical School, University of Sao Paulo.

### Participants

**Fig. 1:**
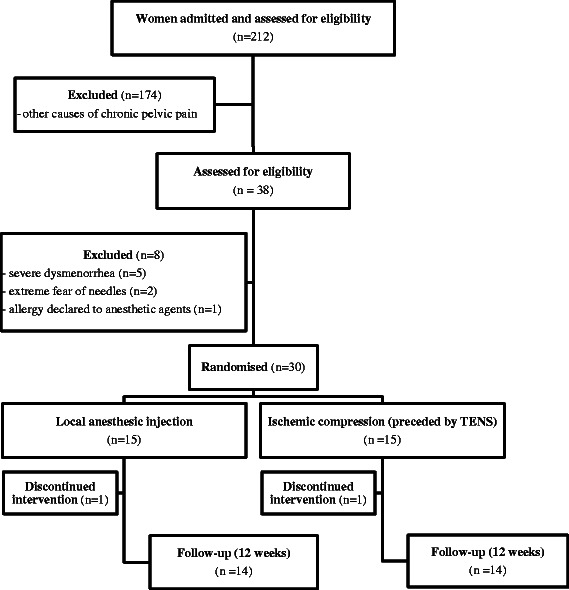
Flowchart of the study

**Fig. 2:**
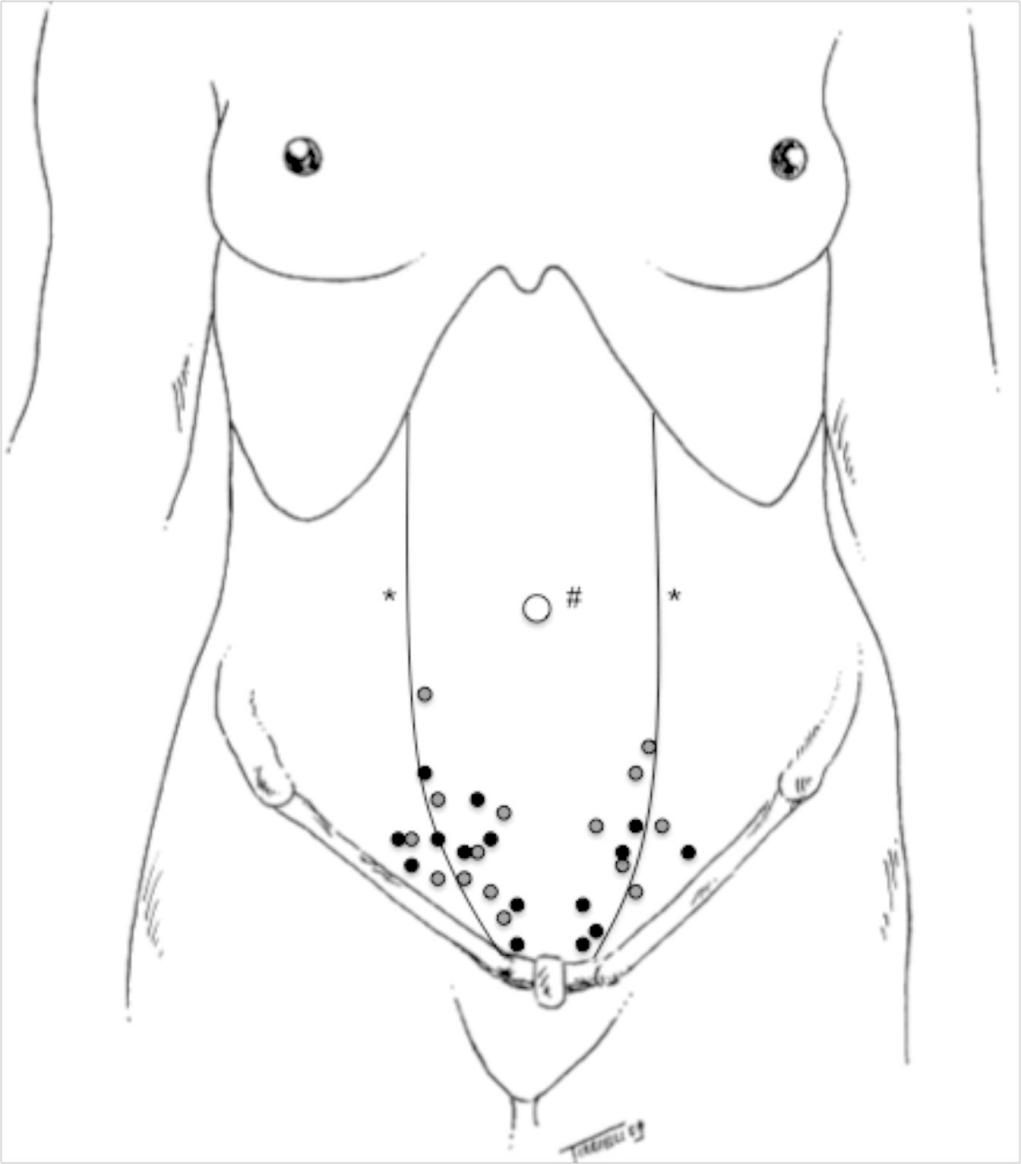
Location of the trigger points. Notes: # umbilical scar; * abdominal rectus edge; dark gray: trigger points from the group 1; soft gray: trigger points from the group 2

Thirty women of reproductive age with CPP and trigger points of the inferior abdominal wall were included in the study. The flow of subjects and location of the trigger points are presented in Figs. 1 and 2, respectively. We excluded women with anticoagulation or bleeding disorders; local or systemic infections; an allergy to anaesthetic agents; acute muscle trauma; extreme fear of needles; a history of chronic musculoskeletal pain complaints such as fibromyalgia, chronic fatigue syndrome, and neurologic or neuropsychiatric conditions; and hypertension or diabetes. We also excluded those who had ingested aspirin within three days of the injection and users of antidepressants or corticosteroids that were administered for at least 30 days. All patients with suspected endometriosis and/or irritable bowel syndrome, interstitial cystitis, and painful bladder syndrome were also excluded. Pelvic/abdominal ultrasound was used to exclude the presence of endometriomas or hernias.

All women were evaluated at baseline and 1, 4, and 12 weeks after the intervention, and were instructed to perform pharmacological washout of central analgesics and/or NSAIDs for at least 72 h prior to all clinical evaluations.

### Interview and examination

All women completed a clinical evaluation. Each woman filled out a form containing the hospital anxiety and depression scale (HAD), visual analogue scale (VAS), McGill Pain Index, and a World Health Organization instrument to assess quality of life (WHOQoL).

### Measurements

Pain threshold (minimal pressure that causes pain or discomfort) and pain tolerance (maximal pressure that the patient can withstand) were measured with an algometer, which is a 1 cm diameter disk attached to the plunger of a pressure gauge. The dial of the gauge is calibrated in kg/cm^2^, with a measuring capacity of 5 kg (Instrutherm Ltda, São Paulo, Brazil). The algometer was placed perpendicularly on the trigger point, and the pressure was increased gradually (0.1 kgf/s) until the patient reported the first painful discomfort (threshold) and until the maximum stimulus withstood (tolerance). Two measurements were made with an interval of three minutes and the mean value was used for analysis.

### Interventions

Group 1: Local anaesthetic injection of 2 mL 0.5 % lidocaine without a vasoconstrictor, directly and perpendicularly applied into the trigger point. No direct pressure was applied after the injection was administered weekly for four weeks.

Group 2: Ischemic compression was applied by sustained pressure on the trigger point. This pressure was sufficient to cause moderate local pain evoking the referred pain pattern [16]. This therapy was applied three times, lasting for 60 s for each session, with a rest period of 30 s between applications [17] for four sessions weekly. The compression was preceded by TENS, which delivers electric stimulation lasting for 30 min using a Dualpex 961 device with a frequency of 100 Hz, pulse of 250 μs, and intensity according to the pain threshold of the patient in order to promote initial analgesia [18].

### Outcomes

Primary outcomes: satisfactory clinical response rate (defined by the Health Ministry of Brazil as a VAS reduction of at least 50 % or as a significant subjective impact on daily life activities (http://bvsms.saude.gov.br/bvs/saudelegis/sas/2012/prt1083_02_10_2012.html).

Secondary outcomes: proportion of pain relief ([VAS before and after treatment]/[VAS before treatment]), threshold, and tolerance on the trigger point.

### Sample size

Sample size estimation was based on the differences between proportions in the satisfactory clinical response rate between the two groups. We considered a 30 % relative change in the between groups rate to be clinically significant. In order to have 90 % power to detect this change with an overall two-sided type I error rate of 5 %, this trial required a total of 60 subjects, or 30 for each group. This sample size was estimated using an online calculator (lee.dante.br).

The trial design included an interim analysis in order to determine if the trial needed to be discontinued early for efficacy or futility. Based on the results of the interim analysis, the study was stopped early, after including five blocks of six subjects each, because we considered the clinical response rate of intervention 2 to be significantly lower than that of intervention 1, after follow-up. At the time of the interim analysis, 15 subjects were enrolled in each group. Based on the O’Brien-Fleming approach group sequential boundary, the significance level for the primary end point was 0.025.

### Randomization

#### Sequence generation

Block randomization, consisting of ten blocks of six subjects each, with three patients in each group of treatment, was generated online (http://www.randomization.com).

#### Control of bias

The first evaluation and the clinical diagnosis were performed by two experts (OBPN, JCRS) using the criteria developed by Travell and Simons [8], in order to recognize active trigger points. A third and fourth researcher, who were blinded to all clinical data except for the location of the trigger point, performed either intervention 1 or 2. An independent observer, who was blind to the previous clinical data and therapy modality, performed the follow-up measurements. A professional who was blinded to all information performed the statistical analysis.

### Statistical analysis

Normal distribution of the data was determined using the Shapiro-Wilk test. Once normal distribution was confirmed, the comparison of quantitative variables between different groups was performed by the *t*-test. When normal distribution was not confirmed, the analysis was performed using the Wilcoxon test. The Chi-square/Fisher’s exact test was used, when appropriate, to analyse nominal variables.

Primary outcome was analysis using generalized linear mixed models. This model was implemented in the SAS program software using the PROC GENMOD. This model estimates the relative risk, independent of time, to verify the relationship between clinical response rate and group. It also estimated the relative risk within each study period. All statistical tests were two-sided. The significance level for the primary end point was adjusted for a single interim analysis to 0.025. Otherwise, we considered P values of less than 0.05 to indicate statistical significance.

In order to compare the times for each group separately, a nonparametric mixed-effects model for longitudinal data [19] was proposed in the analysis of the secondary endpoints. To compare the times for each group separately, we can apply multiple comparisons with Bonferroni adjustment. To compare the groups at each time point separately, the Mann–Whitney test, (a nonparametric technique), was used to compare distributions of two or more groups that require no assumptions about the distribution of data. The level of significance was considered as 5 %. The analysis was conducted by the package nparLD using the R software. Additional file 1: Supplementary material is disponible.

## Results

### Participant flow

The numbers of participants who were assigned, received intended treatment, and were analysed for the primary outcome are presented in Fig. 1. A total of 212 women with CPP were screened at the hospital for study eligibility. One hundred seventy-four cases were excluded after other diagnostic exams for CPP were performed. Eight women with severe dysmenorrhea were excluded due suspected endometriosis. Two patients only attended the first treatment session. Telephone, letter, and e-mail contact was attempted without success. Nevertheless, these patients were included in the analysis.

### Recruitment

Recruitment was performed between February 2008 and March 2010, and patients were followed for 12 weeks. The trial was stopped early because we observed that the clinical response rate was significantly better in one of the arms. The local ethics committee and clinical staff recommended discontinuation of the study.

### Baseline data

Table 1 shows the baseline demographic and clinical characteristics of each group.

**Table 1:** Baseline demographic and clinical characteristics for each group

Variables	Group 1 (*n* = 15)	Group 2 (*n* = 15)	*p*
Age, y [mean ± sd]	38.5 ± 2.8	36.8 ± 3.2	.132
BMI, Kg.m^−2^ [mean ± sd]	28.8 ± 4.1	25.0 ± 3.8	.129
Parity [median, range]	1 (0–6)	1 (0–3)	.700
Abdominal surgeries [median, range]	1 (0–4)	1 (0–3)	820
Measures pretreatment			
VAS, mm [median, interquartile]	54 (49–90)	67 (56–75)	.505
McGill [median, interquartile]	29.5 (13–38)	35.5 (21–42)	.434
Time of symptoms [median, interquartile]	31 (8–76)	38 (9–63)	.331
Pain threshold, kg.cm^−2^ [mean ± sd]	1.05 ± 0.49	1.04 ± 1.30	.988
Pain tolerance, kg.cm^−2^ [mean ± sd]	1.90 ± 0.72	1.81 ± 1.62	.864
HAD anxiety [median, interquartile]	11 (8–16)	13 (10–17)	.187
HAD depression [median, interquartile]	10.5 (5–14)	10.5 (8–13)	.853
WHOQoL [mean ± sd]	53.0 ± 10.3	49.2 ± 14.2	.448

Notes. Group 1: local anesthetic injection; Group 2: ischemic compression via physical therapy; *y* years, *sd* standard deviation; interquartile: 25–75 %, *BMI* body mass index; time of symptoms in months, *HAD* hospital anxiety depression scale, *WHOQoL* World Health Organization quality of life

### Numbers analysed

#### Satisfactory clinical response rate

Clinical response rates were better for the local anaesthetic injection than for ischemic compression (80.0 % (12/15) vs. 40.0 % (6/15) (*p* = 0.018) 1 week after treatment; 80.0 % (12/15) vs. 40.0 % (6/15) (*p* = 0.018) 4 weeks after treatment; and 73.3 % (*n* = 11/15) vs. 13.3 % (2/15) (*p* = 0.00006) 12 weeks after treatment). The estimated relative risk showed that the anaesthetic injection demonstrated approximately 3.8 times better clinical response rates compared to ischemic compression, independent of time (Table 2).

**Table 2:** Estimated relative risk between groups within each time and independent of time (adjust)

Time	Group 1 (*n* = 15)	Group 2 (*n* = 15)	RR crude (IC 95 %)	RR adjust (IC 95 %)^a^
1st week	12 (80,00 %)	6 (40,00 %)	2 (1,02; 3,91)	3,8 (1,17; 8,08)
4th week	12 (80,00 %)	6 (40,00 %)	2 (1,02; 3,91)	
12th week	11 (73,33 %)	2 (13,33 %)	5,5 (1,46; 20,71)	

^a^Relative risk (RR) adjusting by generalized linear mixed models

#### Pain relief

The pain relief of women treated with local anaesthetic injections progressively improved 1, 4, and 12 weeks after intervention. In contrast, women treated with ischemic compression did not show a considerable change in pain relief after intervention (Table 3). The percent rate of improvement (1, 4 and 12 weeks after intervention) was progressively higher in group 1 (reduction of 45.3, 60.2 and 69.9 % of VAS, respectively) (*p* = 0.03) compared to group 2 (reduction of 18.7 %, 9.2 %).

**Table 3:** Evaluation of percentage of pain relief between groups and intra group (between different times of follow-up)

Groups	VAS Baseline % [median(range)]	VAS 1st week % [median(range)]	VAS 4th week % [median(range)]	VAS 12th week % [median(range)]	P^d^
Group 1	0.0 [54 (49–90)]	−45.3^a^ [30.5 (8–58)]	−60.2^b^ [10 (0–49)]	−69.9^c^ [8 (0–39)]	.03
Group 2	0.0 [67 (56–75)]	−18.7^a^ [50 (47–54)]	−9.2^b^ [60 (42–61)]	−8.5^c^ [56 (42–67)]	.47
P	.50	.08	<.01	<.01	---

Notes: all measurements are represented by percentage of pain relief (%VAS) and by median and 25–75 % quartiles (range) of VAS. The minus signal (−) signify reduction of pain scores. Post test for threshold at Group 1: ^a^ vs ^b^ = .06; ^a^ vs ^c^ = .01; ^b^ vs ^c^ = .10; ^d^ time effect

Although the differences between groups in the various time points were not significant, pain threshold and tolerance improved progressively in the women who received the local anaesthetic injection (Table 4).

**Table 4:** Evaluation of measurements of pain threshold and pain tolerance between groups and intra group (between different times of follow-up)

Groups	Baseline	1st week	4th week	12th week	P^d^
Threshold					
Group 1	1.00 (0.85–1.31)	0.97^a^ (0.88–1.04)	1.44^b^ (1.08–1.62)	1.37^c^ (1.26–1.49)	<.01
Group 2	0.72 (0.57–1.06)	1.21 (0.84–1.82)	1.08 (0.92–1.13)	1.10 (0.95–1.27)	.76
*p*	.99	.32	.13	.97	
Tolerance					
Group 1	1.87 (1.42–2.24)	1.60^a^ (1.46–1.72)	2.00^b^ (1.73–2.23)	2.23^c^ (2.22–2.24)	<.01
Group 2	1.44 (1.04–1.92)	1.91 (1.53–2.44)	1.51 (1.22–1.87)	1.46 (1.32–1.49)	.52
*p*	.86	.67	.09	.24	

Notes: all measurements are represented by median and 25–75 % quartiles. Post test for threshold at Group 1: ^a^ vs ^b^ < .01; ^a^ vs ^c^ < .01; ^b^ vs ^c^ = .02; Post test for tolerance at Group2: ^a^ vs ^b^ < .03; ^a^ vs ^c^ < .01; ^b^ vs ^c^ = .67; ^d^time effect

#### Harmful or unintended effects

There were no important harmful or unintended effects. However, two patients in Group 1 (local anaesthetic injection) presented with ecchymoses (3.4 cm and 5.1 cm of extension) that resolved spontaneously within 4 and 6 weeks, respectively.

## Discussion

### Synthesis

In the present study, we observed that local anaesthetic injections were superior to ischemic compression, and resulted in progressive improvements in pain relief. Despite the absence of statistical differences with the compression group, we observed that the local pain threshold and tolerance of women submitted to a local anaesthetic injections improved throughout the follow-up period and until the end of treatment.

### Interpretation

To our knowledge, this is the first randomized trial demonstrating the superiority of a local anaesthetic for the treatment of the trigger points in the inferior abdominal wall of women with chronic pelvic pain even though this method has been proven to be effective in the treatment of other myofascial syndromes [20], and is similar to the effects of the lidocaine patch [21].

The effects of acute anaesthesia that are promoted by local anaesthetics are well known, and are believed to occur by interrupting nerve excitation and conduction by direct interaction with voltage-gated Na channels. Reduction of inflammation and activation TRPV1 e TRPA1 might explain long term effects of lidocaine [22]. However, we observed that, even after the injections were stopped, the pain relief reported by the subjects who received this intervention was significantly progressive. The present study does not permit us to reach precise conclusions about the mechanisms associated with this continued long-term effect. It is likely that this effect can be explained by the affinity of lidocaine with the Na channel, since it is known that the drug has a low affinity for the channels, when they are in the standby mode and demonstrated higher affinity when they are open and/inactivated [23]. The application of anaesthetics may be associated with the occurrence of skeletal muscle tissue or neural damage [24]. Nevertheless, muscle injury is usually reversible and tissue regeneration occurs within 4 to 6 weeks [25]. Although neurotoxicity may justify the progressive effect of local anaesthetic injections, we did not identify other clinical signs during the study period, such as dysesthesia, paresthesia, or sensorimotor deficits that differ from those reported at baseline [26]. Although we tend to associate pain relief to the presence of a local anaesthetic, we cannot overlook the potential effect of dry needling, it has been shown to be capable to evoking antinociceptive effects by segmental modulation. Although this effect seems to be considerable for a few minutes [27], it could not completely explain our results. Nevertheless, the effectiveness of the dry needle seems to depend on the integrity of the afferent and spinal cord circuitry [28], which may involve supraspinal pain control via midbrain periaqueductal grey activation, among others. Some studies have demonstrated that this is an effective and safe method for the treatment of trigger points [29], which may present with effects similar to placebo [30].

In contrast, even though we had observed a considerable improvement of pain at the trigger points immediately after the ischemic compression (preceded by TENS), this improvement was not maintained until follow-up. Recent literature studies have demonstrated changes in blood flow and cell metabolism at a myofascial trigger point after release with ischemic compression [31], and improvement of pain secondary to myofascial syndromes after ischemic compression [32]. However, the studies did not involve sufficiently long follow-up times for the clearance of the placebo effects of the method. TENS, in turn, has shown positive short-term effects on trigger points, but not during medium or long-term follow-up [33]. At present, we understand that it is not possible to recommend ischemic compression as a first-line therapeutic modality for the treatment of trigger points of the abdominal wall.

### Limitations

Although the present results demonstrate the superiority of one of the methods, we have to recognize that the early interruption of the trial is a limiting factor. This may increase the possibility of an alpha error, which favour the overestimation of the effect. Although currently available empirical evidence suggests that early interrupted clinical trials overestimate the effects of new treatments and that the reasons used to justify interrupting the trial are often not sufficiently specified [34], after a judicious evaluation by our physician and the ethics committee staff, we believe that interrupting the trial was an ethical obligation.

## Conclusions

The results of the present study suggest that local anaesthetic injection is superior to ischemic compression for the treatment of abdominal wall trigger points. Since we selectively excluded subjects with comorbidities and other causes of chronic pelvic pain, we cannot confirm that this intervention, compared to those that had been applied by experts, works in the more complex “real-life” setting. Thus, future pragmatic clinical trials are needed in order to confirm the effectiveness of this method in a wider variety of circumstances as well as assess whether it is better compared to techniques such as dry needling or acupuncture, for example.

## Additional file

Additional file 1: **Supplementary material.** (DOCX 493 kb)

## Abbreviations

CPP: chronic pelvic pain
HAD: hospital anxiety and depression scale
NSAID: non-steroidal anti-inflammatory drugs
TENS: transcutaneous electric nervous stimulation
VAS: visual analogue scale
WHOQoL: World Health Organization instrument to assess quality of life
