# Supplementary material for: Anaesthetic injection versus ischemic compression for the pain relief of abdominal wall trigger points in women with chronic pelvic pain
Source: BMC Anesthesiol. 2015 Dec 1;15:175. doi: 10.1186/s12871-015-0155-0 (PMC4667406; doi:10.1186/s12871-015-0155-0)
Supplement: Supplementary file 1 — Supplementary material. (DOCX 493 kb) [file 12871_2015_155_MOESM1_ESM.docx]

Additional file 1: Supplementary material

data dados;

input ind grupo tempo resp;

cards;

1 1 1 1

2 1 1 1

3 1 1 1

4 1 1 1

5 1 1 1

6 1 1 1

7 1 1 1

8 1 1 0

9 1 1 1

10 1 1 1

11 1 1 1

12 1 1 1

13 1 1 1

14 1 1 0

15 1 1 0

16 2 1 1

17 2 1 0

18 2 1 0

19 2 1 0

20 2 1 0

21 2 1 0

22 2 1 0

23 2 1 0

24 2 1 1

25 2 1 1

26 2 1 0

27 2 1 1

28 2 1 1

29 2 1 1

30 2 1 0

1 1 2 1

2 1 2 1

3 1 2 1

4 1 2 1

5 1 2 1

6 1 2 1

7 1 2 1

8 1 2 0

9 1 2 1

10 1 2 1

11 1 2 1

12 1 2 1

13 1 2 1

14 1 2 0

15 1 2 0

16 2 2 1

17 2 2 0

18 2 2 0

19 2 2 0

20 2 2 0

21 2 2 0

22 2 2 0

23 2 2 0

24 2 2 1

25 2 2 1

26 2 2 0

27 2 2 1

28 2 2 1

29 2 2 1

30 2 2 0

1 1 3 1

2 1 3 0

3 1 3 1

4 1 3 1

5 1 3 1

6 1 3 1

7 1 3 1

8 1 3 0

9 1 3 1

10 1 3 1

11 1 3 1

12 1 3 1

13 1 3 1

14 1 3 0

15 1 3 0

16 2 3 0

17 2 3 0

18 2 3 0

19 2 3 1

20 2 3 0

21 2 3 0

22 2 3 0

23 2 3 0

24 2 3 0

25 2 3 0

26 2 3 0

27 2 3 1

28 2 3 0

29 2 3 0

30 2 3 0

;;

proc print;

run;

proc freq;

where tempo=1;

table grupo*resp / nopercent nocol relrisk;

run;

proc freq;

where tempo=2;

table grupo*resp / nopercent nocol relrisk;

run;

proc freq;

where tempo=3;

table grupo*resp / nopercent nocol relrisk;

run;

proc genmod ;

class tempo grupo ind;

model resp= tempo grupo grupo*tempo / dist=binomial link=log;

repeated subject=ind / type=cs corrw covb;

estimate "grupo" grupo -1 1 / exp;

run;
